# Self-rated resilience and mobility limitations as predictors of change in active aging during COVID-19 restrictions in Finland: a longitudinal study

**DOI:** 10.1007/s10433-021-00634-6

**Published:** 2021-08-28

**Authors:** Sini Siltanen, Erja Portegijs, Milla Saajanaho, Katja Pynnönen, Katja Kokko, Taina Rantanen

**Affiliations:** 1 Finnish Institute for Health and Welfare, PO Box 30, 00271 Helsinki, Finland; 2 Gerontology Research Center and Faculty of Sport and Health Sciences, University of Jyväskylä, Jyväskylä, Finland

**Keywords:** Social distancing, Participation, Walking difficulty, Coping, Older people

## Abstract

Social distancing during the COVID-19 pandemic decreased older people’s opportunities to lead an active life. The purpose of this study was to investigate whether walking difficulties predict changes in leading an active life during the COVID-19 social distancing recommendation compared to 2 years before, and whether self-rated resilience moderates this association among older people. Data were collected during social distancing recommendation in May and June 2020 and 2 years before (2017–18) among community-living AGNES study participants initially aged 75, 80, or 85 years (*n* = 809). Leading an active life was assessed with the University of Jyväskylä Active Aging Scale (UJACAS; total score range 0–272) and resilience with the 10-item Connor-Davidson Resilience Scale (0–40). Self-reported walking difficulties over a 2 km distance were categorized into no difficulty, difficulty, and unable to walk. The total UJACAS score declined 24.9 points (SD 23.5) among those without walking difficulty, 27.0 (SD 25.0) among those reporting walking difficulty and 19.5 (SD 31.2) among those unable to walk 2 km. When adjusted for baseline UJACAS score, those unable to walk 2 km demonstrated the greatest decline. Baseline resilience moderated this association: Higher resilience was associated with less declines in UJACAS scores among persons with or without walking difficulty, and with more declines among persons unable to walk 2 km. When opportunities for leading an active life are compromised, those with less physical and psychological resources become particularly vulnerable to further declines in activity.

## Introduction

During spring 2020, the coronavirus disease, COVID-19, outbroke globally. To protect populations’ health and the functioning of societies and economy, in many countries, public gatherings were limited to few people, unnecessary traveling was prohibited, and, e.g., libraries, museums, leisure centers, and sport facilities were closed down (WHO 2020; Finnish Government Communications Department, Press Release 140/2020). The Government in Finland declared a state of emergency in March 2020. In addition to restricting the availability of activities in the community, a general guideline was laid out according to which all people aged over 70 years should adopt quarantine-like conditions, i.e., avoid physical contact with others outside one’s household (Finnish Government Communications Department, Press Release 140/2020).

During the COVID-19 restrictions, in the early phase of the pandemic, many forms of out-of-home mobility decreased (Sepúlveda-Loyola et al. 2020; Rantanen et al. 2020). However, physical activity, such as walking for fitness, increased among many Finnish community-dwelling older people, because it was one of the few available hobbies that could spontaneously be done (Rantanen and Portegijs 2020; Portegijs et al. 2021). It is likely that increases in such physical activities were possible only for people with intact mobility.

We recently reported that leading an active life declined during the social distancing recommendations among community-living older people (Rantanen et al. 2020). Active life reflects an individual’s striving to engage in various activities of choice as per one’s goals, functional capacities, and overall opportunities (Rantanen et al. 2019). We refer to this as active aging at the level of individuals. It covers all forms of doing, not just participation in physical activities, and is an important correlate of well-being (Rantanen et al. 2018, 2020). Active aging, as it manifests in individuals’ lives, has not been widely studied, because a validated quantitative instrument to assess it became available only recently (Rantanen et al. 2018). The University of Jyväskylä Active Aging Scale (UJACAS) includes 17 activities defined based on their meaning to an individual rather than external criteria (e.g., enjoying the outdoors, maintaining social relationships, making home cozy, or taking care of one’s appearance). For each activity the will to act, the ability and opportunity to act and the extent of doing the activity are assessed, and the scores are summed.

Cross-sectionally, more severe mobility limitations and lower resilience, previously referred to as self-rated coping abilities, correlate with lower scores (Siltanen et al. 2020). However, during COVID-19 restrictions, opportunities for engaging in different activities were limited for all. It is unknown whether active aging scores declined similarly regardless of physical and psychological resources, or whether those with less physical or psychological resources showed more or less extensive changes considering that their ‘starting points’ differed. In addition, it is unclear whether all active aging components, i.e., will to act, ability to act, opportunity to act, and actual activity, were equally affected by the social distancing recommendations.

Walking difficulties over longer distances are typically the first signs of functional decline among older people (Mänty et al. 2007; Verbrugge and Jette 1994). Walking difficulties make it more burdensome to engage in activities outside home. Eventually, they may evolve to inability and increase the risk of dependency for out-of-home activities, and potentially, gradually lead to giving up on activities considered too arduous. In a cross-sectional setting, walking difficulties were associated with a lower level of active aging, whereas high self-rated resilience was associated with a higher level, thereby compensating at least partly for physical decline (Siltanen et al. 2020). Resilience refers to the ability to adapt positively to adversity (Dyer and McGuinness 1996). The common perception is that resilience, or the attainment of positive adjustment, can only be manifested when a person is exposed to a significant threat or hardship, for example, a global COVID-19 pandemic or mobility decline. Resilience may partially explain why individuals respond differently to these challenges, as individuals’ with higher resilience are prone to look to the future, solve problems effectively (Van Kessel 2013), and compensate for deficits in function (Carpentieri et al. 2017; Siltanen et al. 2020), i.e., utilize various coping strategies.

We expected that higher resilience may prevent or slow down the decline in active aging during social distancing. When facing challenges, people with higher resilience typically stay tenacious and persistent with their pursuits, but at the same time, may show flexibility by downgrading the importance of goals no longer feasible, and start to pursue other more feasible objectives (Brandtstädter and Renner 1990). We have previously shown that when facing mobility limitations, people with higher resilience can modify their activity by substituting out-of-home or social activities by at-home or solitary activities that are still possible to perform (Siltanen et al. 2020). We anticipated that similar modifications may have taken place also during the COVID-19 social distancing, when many destinations of interest or leisure activities in the community were suspended, and avoiding physical contact was recommended.

The purpose of this study was to investigate whether 2 km walking difficulty ascertained prior to the COVID-19 pandemic predicted changes in active aging total score and its subscores during social distancing recommendations compared to 2 years before. We also studied whether self-rated resilience moderated these changes.

## Methods

### Data

The present study forms part of the Active Aging—Resilience and External Support as Modifiers of the Disablement Outcome (AGNES) study project. The protocol, study design, and recruitment of the AGNES baseline study have been presented in detail elsewhere (Rantanen et al. 2018). Briefly, AGNES was a population-based cohort study comprising community-dwelling people from three birth cohorts initially aged 75, 80, or 85 years and residing in the city of Jyväskylä in Central Finland. Baseline data were collected with home-interviews from 1018 participants between September 2017 and December 2018 (Portegijs et al. 2019).

The AGNES COVID-19 follow-up was conducted in May and June 2020 during the self-quarantine recommendations, which took place between March 13th and June 23rd. Average follow-up time was 2 years (range 1.5 to 2.7 years). The recruitment has been described earlier (Rantanen et al. 2020)*.* Briefly, all baseline participants who were not known to have moved to an institutional care facility or died and who had not withdrawn their consent for study participation were invited to participate in the AGNES COVID-19 survey (*n* = 985). The response rate was 82% (*n* = 809). Reasons for not participating were unwillingness to take part (*n* = 127), being out of reach (*n* = 30), and death (*n* = 4). In addition, 15 persons were excluded because they were unable to respond or they had moved to an assisted living facility (Rantanen et al. 2020*)*. Due to the social distancing guidelines, the follow-up data were collected with postal questionnaires (or phone interviews, *n* = 7, in case the participant had trouble answering the questionnaire). As reported earlier, participants in the AGNES COVID-19 survey were somewhat younger, healthier, and better functioning than those who only took part in the AGNES baseline study (Rantanen et al. 2020).

### Ethics

The Central Finland Hospital District approved the initial AGNES study protocol on August 23, 2017 and gave an additional positive ethical statement for the COVID-19 follow-up study on May 13, 2020. All participants signed written consents before the baseline assessments and consented to the follow-up by returning the postal questionnaire. The study protocol followed the principles declared by the Declaration of Helsinki.

### Study variables

Leading an active life was measured with the validated University of Jyväskylä Active Aging Scale (UJACAS) (Rantanen et al. 2019). The active aging scale comprises 17 items, such as practicing memory, enjoying the outdoors, helping others, exercising, maintaining friendships, crafting or DIY, advancing societal/communal matters, and making one’s days interesting. Each activity is assessed from four aspects forming the subscores: will to act, ability to act, opportunity to act, and frequency or extent of doing the activity. Participants are asked to consider the past 4 weeks while answering. Response options range on a five-point Likert scale from 0 (lowest, e.g., not at all) to 4 (highest, e.g., almost daily or very strongly). First, we calculated the four subscores (0–68) by summing the item scores, and subsequently, the total active aging score (0–272) by summing the subscores. Higher values indicate that people lead a more active life.

Walking difficulties over a 2 km distance were self-reported and assessed at baseline. The response options were “able without difficulty,” “able with some difficulty,” “able with a great deal of difficulty,” “unable without the help of another person,” and “unable even with help.” We formed three categories according to the degree of walking difficulty (1) no difficulty, (2) (at least some) difficulty, and (3) unable to walk (independently or even with assistance). Those who reported having no difficulty in walking 2 km formed the reference category. This self-report question has been shown to be valid and reliable (Mänty et al. 2007).

Self-rated resilience was assessed at baseline with a modified 10-item version of the Connor-Davidson Resilience Scale (CD-RISC10) (Campbell-Sills and Stein 2007), instructing participants to consider their life in general rather than just the past 4 weeks. The CD-RISC10 scale includes items such as “can deal with whatever comes,” “can achieve goals despite obstacles,” and “not easily discouraged by failure,” and thus reflects the respondent’s perception of his/her ability to cope with and bounce back from various stressful situations. Response options follow a five-point Likert scale ranging from 0 (not true at all) to 4 (true nearly all of the time). We calculated a sum score (0–40) when at least seven items received a response. Single missing items were imputed for 12 participants based on the mean of the responses they provided to the other items. We consider that higher scores indicate higher self-reliance in one’s ability to cope with different adversities of life. The CD-RISC10 has shown good validity and acceptable reliability in the AGNES baseline sample (Tourunen et al. 2019).

Descriptive variables for baseline group comparisons included age, sex, number of years of education, living alone, cognitive performance, and number of chronic diseases. Data on age and sex were included in the sampling by the Population Information System of Finland. Years of education were self-reported. Living alone was assessed with question “Who do you live with?” and the responses were categorized into “alone” vs. “not alone.” Cognitive performance was measured with the Mini Mental State Examination (MMSE), which assesses global cognition (Folstein et al. 1975). Number of physician-diagnosed chronic conditions was self-reported and calculated based on a list of 34 common conditions and an additional open-ended question (Rantanen et al. 2018).

### Statistical methods

Background characteristics of study participants by baseline walking difficulty category were described with means and standard deviations (SD) or percentages. Differences between groups were tested with one-way ANOVA (continuous variables) and chi-square test (categorical variables). Changes in active aging scores were computed by subtracting the baseline score from the follow-up score. Correlations between baseline resilience and these change scores were calculated with Pearson’s correlation. In addition, whether the mean changes in active aging scores differed between walking difficulty categories was tested with one-way ANOVA and Bonferroni post hoc test. Finally, to study how walking difficulties and resilience are associated with active aging and its four aspects at follow-up, we performed ordinary least squares (OLS) regression analyses. First, we added resilience and walking difficulty as independent variables in separate models. Thereafter, they were added to the model simultaneously and allowed to interact to find out whether coping ability moderates the association between walking difficulty and active aging. The moderation effect was probed using regression centering with the 16th, 50th, and 84th percentiles of the distribution of the coping ability scale describing relatively low, moderate, and relatively high values. The regression analyses were performed separately for all active aging scores. The models were first adjusted for the respective baseline active aging score, and thereafter, additionally for age and sex. Significance level was set at .05, and all analyses were conducted with SPSS version 26. The PROCESS macro for SPSS by Andrew Hayes (2017) was utilized to conduct the moderation analyses.

## Results

### Descriptives

Baseline characteristics of study participants by baseline walking ability are presented in Table 1. On average, those with walking difficulties or unable to walk the 2 km distance were older, had more chronic conditions and poorer self-rated ability to cope with adversity, and a greater proportion of them were women and living alone compared to those with no walking difficulty. In addition, those reporting inability to walk had poorer cognitive performance and fewer years of education than those with no walking difficulty.

**Table 1:** Baseline characteristics of study participants by walking ability category

	All participants	2 km walking ability			*p*^a^
	(*n* = 789)	No difficulty (*n* = 525)	Difficulty (*n* = 212)	Unable (*n* = 52)	
	Mean ± SD	Mean ± SD	Mean ± SD	Mean ± SD	
Cognitive function (MMSE)	27.5 ± 2.2	27.6 ± 2.1	27.4 ± 2.1	26.1 ± 2.7	< .001
Number of chronic conditions	3.3 ± 2.0	2.9 ± 1.7	4.3 ± 2.1	5.0 ± 2.5	< .001
Years of education	11.8 ± 4.8	12.0 ± 4.3	11.8 ± 5.7	10.1 ± 4.5	.024
Coping ability (CD-RISC10)	31.3 ± 5.1	31.6 ± 4.9	30.4 ± 5.5	29.6 ± 5.9	< .001

	%	%	%	%	*p*^b^
Women	59	54	69	65	< .001
Age					< .001
75 years	48	55	36	33	
80 years	32	31	37	23	
85 years	20	14	27	44	
Living alone	40	33	51	67	< .001

*MMSE* Mini Mental State Examination, *CD-RISC10* 10-item Connor-Davidson Resilience Scale

^a^Tested with one-way ANOVA

^b^Tested with chi-square test

Table 2 shows that all active aging scores at baseline were highest among those with no walking difficulty and lowest among those unable to walk 2 km, and people with walking difficulty formed the middle group. Of the subscores, the greatest decline from baseline to follow-up was observed for the opportunity to act. However, differences between walking categories were only observed for the will to act subscore, and only between those unable to walk 2 km, and those with walking difficulty, who experienced a greater decline, as pointed out in a Bonferroni post hoc test. Resilience at baseline had a statistically significant, but weak negative correlation with change in will to act subscore (*Pearson’s r* = − .09, *p* = .014) and ability to act subscore (*Pearson’s r* = − .08, *p* = .032), meaning that the poorer the self-reliance in one’s ability to cope with different challenges in life, the more the will and ability to act subscores declined.

**Table 2:** Mean baseline scores and changes in active aging during the 2-year follow-up by 2 km walking ability category

UJACAS score (range)	2 km walking ability			*p*
	No difficulty (*n* = 505)	Difficulty (*n* = 203)	Unable (*n* = 50)	
	Mean ± SD	Mean ± SD	Mean ± SD	
Total (0–272)				
Baseline	202.4 ± 26.5	186.0 ± 31.1	150.9 ± 33.6	< .001
Change	− 25.0 ± 23.5	− 27.2 ± 25.0	− 20.0 ± 31.2	.157
Will to act (0–68)				
Baseline	44.7 ± 9.0	43.0 ± 9.8	37.8 ± 10.6	< .001
Change	− 5.5 ± 8.0	− 6.8 ± 7.4	− 3.9 ± 9.6	.034
Ability to act (0–68)				
Baseline	62.1 ± 6.4	55.7 ± 7.9	45.2 ± 10.0	< .001
Change	− 4.0 ± 7.5	− 4.4 ± 8.3	− 4.5 ± 9.9	.741
Possibility to act (0–68)				
Baseline	53.6 ± 8.8	48.2 ± 10.1	36.7 ± 11.2	< .001
Change	− 10.3 ± 9.6	− 10.4 ± 9.7	− 7.4 ± 10.5	.115
Amount of activity (0–68)				
Baseline	41.9 ± 8.1	39.1 ± 9.1	31.2 ± 9.5	< .001
Change	− 5.1 ± 7.6	− 5.8 ± 7.4	− 3.9 ± 8.4	.233

*UJACAS* University of Jyvaskyla Active Aging Score, *SD* standard deviation

*p* value tested with one-way ANOVA

### OLS analyses

The OLS analyses (Table 3) showed that when adjusting for baseline differences in UJACAS total score, the UJACAS total score decline over time was steeper among those with walking difficulty and those unable to walk 2 km decline than those without walking difficulty. The changes in UJACAS subscores were parallel to changes in total score for those with no walking and walking difficulty, but among those unable to walk 2 km, the results were more diverse (Table 3). Regarding the will to act subscore, those unable to walk 2 km did not differ from those without walking difficulty, but the decline in ability to act subscore was substantially greater among those unable to walk 2 km than among those without walking difficulty.

**Table 3:** Associations of coping ability and walking difficulty with active aging (UJACAS total score) at follow-up tested with OLS regression analysis

UJACAS score	*B*	SE	*p*
Total			
Model 1			
Coping ability	.26	.19	.168
Model 2			
No walking difficulty	0.0	Ref.	Ref.
Walking difficulty	− 6.54	2.00	.001
Unable to walk	− 8.81	3.79	.020
Model 3			
No walking difficulty * coping	0.0	Ref.	Ref.
Walking difficulty * coping	− .03	.39	.945
Unable to walk * coping	− 1.21	.61	.047
Will to act			
Model 1			
Coping ability	.07	.06	.192
Model 2			
No walking difficulty	0.0	Ref.	Ref.
Walking difficulty	− 1.89	.61	.002
Unable to walk	− .93	1.10	.398
Model 3			
No walking difficulty * coping	0.0	Ref.	Ref.
Walking difficulty * coping	− .02	.12	.879
Unable to walk * coping	− .26	.19	.171
Ability to act			
Model 1			
Coping ability	.08	.06	.166
Model 2			
No walking difficulty	0.0	Ref.	Ref.
Walking difficulty	− 2.15	.69	.002
Unable to walk	− 5.27	1.34	< .001
Model 3			
No walking difficulty * coping	0.0	Ref.	Ref.
Walking difficulty * coping	− .13	.13	.320
Unable to walk * coping	− .47	.20	.018
Possibility to act			
Model 1			
Coping ability	.15	.07	.029
Model 2			
No walking difficulty	0.0	Ref.	Ref.
Walking difficulty	− 2.18	.76	.004
Unable to walk	− 3.99	1.44	.006
Model 3			
No walking difficulty * coping	0.0	Ref.	Ref.
Walking difficulty * coping	.09	.15	.564
Unable to walk * coping	− .20	.23	.383
Amount of activity			
Model 1			
Coping ability	.15	.05	.005
Model 2			
No walking difficulty	0.0	Ref.	Ref.
Walking difficulty	− 1.67	.58	.004
Unable to walk	− 2.67	1.08	.014
Model 3			
No walking difficulty * coping	0.0	Ref.	Ref.
Walking difficulty * coping	.08	.12	.486
Unable to walk * coping	− .42	.18	.022

*UJACAS* University of Jyvaskyla Active Aging Score, *OLS* ordinary least squares. All models were adjusted for respective baseline UJACAS score. *B* unstandardized regression coefficient, *SE* standard error

Resilience was associated with the opportunity to act and amount of activity subscores (Table 3). The regression coefficients were positive, although low, meaning that higher self-reliance in one’s ability to cope with different challenges in life at baseline predicted higher scores in the opportunity to act and activity subscales at follow-up. Adjusting the models additionally for age and sex did not alter the results markedly (*data not shown*).

**Fig. 1:**
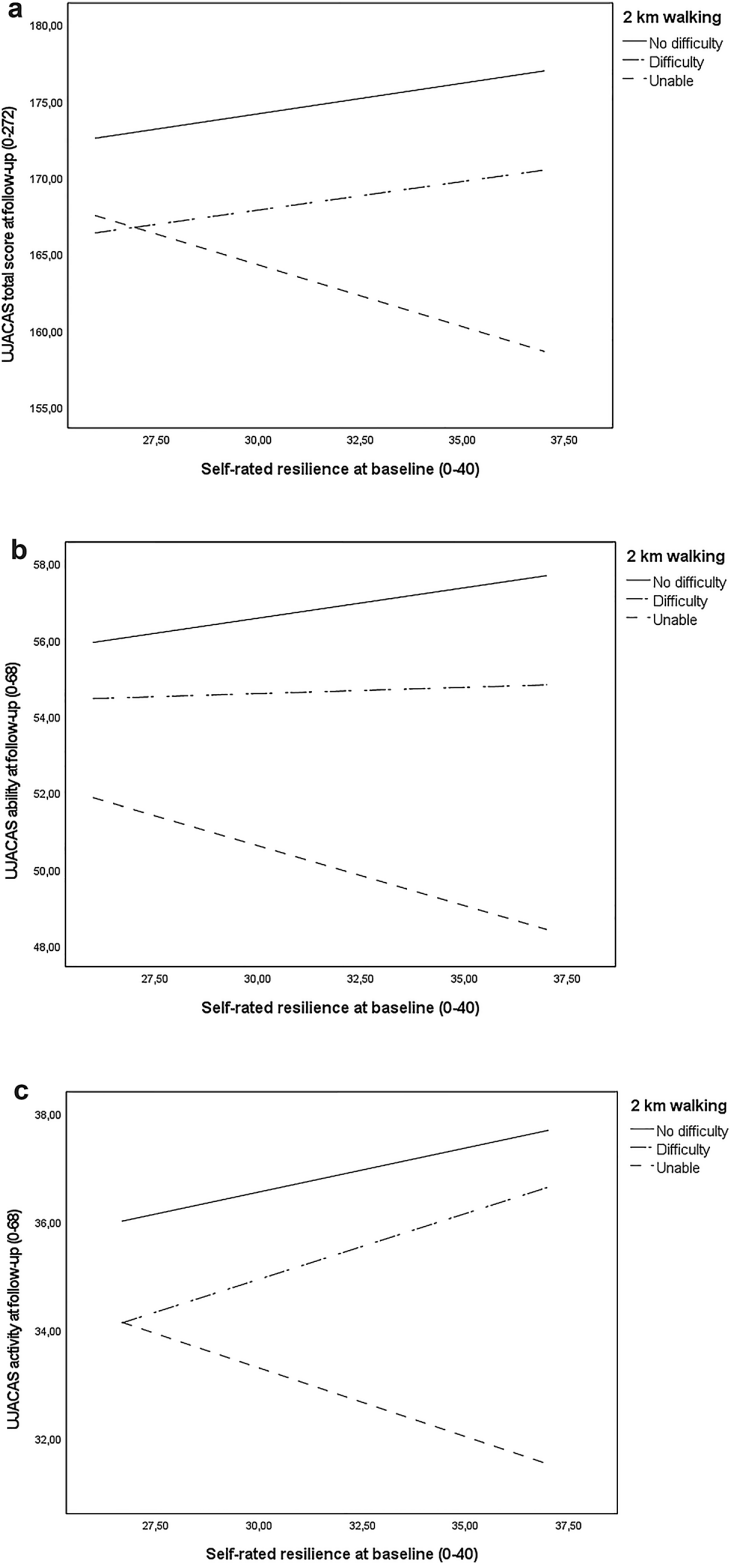
Illustration of the OLS path analysis with coping ability (CD-RISC10) as a moderator of the association between baseline 2 km walking difficulties (reporting walking difficulty or being unable to walk independently vs. reporting no difficulty) and **a** active aging total score (*n* = 729), **b** ability to act subscore (*n* = 744), and **c** amount of activity subscore (*n* = 747) at follow-up

Finally, as a third step, we tested whether resilience moderated the associations between UJACAS score changes and walking difficulty. Statistically significant interactions were observed for UJACAS total score, ability to act subscore and activity subscore (Table 3). Among persons with no difficulty and those with walking difficulty, higher resilience at baseline was associated with higher UJACAS total score at follow-up. In contrast, among persons unable to walk 2 km, higher resilience was associated with lower UJACAS total score at follow-up (Fig. 1a). This moderation effect of resilience was similar also for the ability to act (Fig. 1b) and activity subscores (Fig. 1c).

## Discussion

Earlier we reported that active aging declined during the social distancing recommendations among Finnish community-living older people (Siltanen et al. 2020). The present study shows that the decline was especially remarkable among persons with pre-existing mobility limitation. Higher resilience, here assessed as self-reliance in one’s ability to cope with different adversities of life and ascertained prior to the pandemic, alleviated the negative effects of the COVID-19-related restrictions and declining mobility on active aging, as persons with higher resilience retained higher active aging scores over the follow-up. This mitigating role of resilience was particularly clear for the active aging total score and the activity subscore. Nevertheless, as suggested previously by our cross-sectional analyses (Siltanen et al. 2020), better self-rated resilience did not slow down but instead intensified active aging decline for persons at a more severe phase of mobility decline. This study contributes to the rather scarce literature on the associations of COVID-19-related social distancing with older people’s everyday behavior, and extends previous knowledge, mainly centered on physical activity (e.g., Yamada et al. 2020a; b; Portegijs et al. unpublished), to a more comprehensive approach to activities of choice among older people.

Decline was observed in all four active aging subscores but it was most pronounced in the opportunity to act subscore, when comparing the situation 2 years before to the situation amid the COVID-19 restrictions. This is logical, because the actions to control the spreading of the Sars-Cov-2 virus included closing down of destinations of interest (e.g., libraries, restaurants, theaters) and suspending most activities where close contact with people outside one’s household could facilitate the viral spreading. This was duly reflected in the responses. The ability to act subscore, in turn, declined markedly among those unable to walk 2 km at the baseline. This implies that the gradually decreasing possibilities to do things among people with more severe mobility limitations were further diminished amid the COVID-19 social distancing. The expected consequences of social distancing, i.e., social isolation and being homebound, synergistically increase the risk of health decline among older people (Sakurai et al. 2019). Moreover, it is worth bearing in mind that the will to engage in different activities among persons unable to walk 2 km did not decline. We have previously studied unmet physical activity need (Rantakokko et al. 2010) and suggest that during social distancing recommendations, people with mobility limitations were experiencing unmet activity needs. This has probably reduced their well-being, because people are naturally motivated to direct their activities to fulfill needs for autonomy, relatedness, and competence (Deci and Ryan 2000), all of which were undermined during social distancing, and especially much among those with mobility limitations. This topic warrants further investigation.

The present findings are in line with our earlier results suggesting that higher resilience compensates for early-phase losses in function and helps older people to maintain their desired activity levels (Siltanen et al. 2020)—even when extreme environmental restrictions take place. People with higher resilience are often tenacious and persistent with their pursuits to achieve personal goals and desires, but also able to adapt to changed circumstances by modifying their goals, for example by switching previous activities into new, more feasible ones (Brandtstädter and Renner 1990). The present participants with higher resilience engaged more frequently in activities that were still available, which is in line with our earlier cross-sectional observations (Siltanen et al. 2020). For example, they participated in many at-home activities, enjoyed the nature, and exercised—even despite walking difficulties. These findings suggest that self-rated resilience may explain the differences in activity that are not accounted for by health and function.

The observation that higher self-reliance in one’s ability to cope with different adversities of life at baseline among people unable to walk 2 km predicted steeper active aging decline over the follow-up can be explained by several ways. First, it is possible that over the follow-up, their mobility decline progressed to worse, and they became more dependent on external help. In the present sample, a greater proportion of those unable to walk 2 km lived alone than of those in the other walking ability categories. Hence, it is likely that they have neither had the resources to go outside themselves nor anyone to assist them in doing so. People with more advanced mobility limitations also typically suffer from other functional and health deficits, such as cognitive decline (e.g., Demnitz et al. 2016), which may not only further reduce their possibilities for active aging but also make them a high-risk group for severe COVID-19 infections. It is possible that due to fear of infection, these people have purposely reduced their activity. Another potential explanation for this finding is that during the COVID-19 restrictions, higher resilience among those unable to walk 2 km mostly presented itself as psychological flexibility. Psychological flexibility refers to a coping strategy in which a person gives up on certain blocked goals or downgrades their importance in order to adapt to changed circumstances and adversity (Brandstädter and Renner 1990, Brandstädter and Rothermund 2002). Eventually, this kind of adaptation, i.e., accepting the current situation, leads to greater well-being, but may manifest as lower active aging scores. Lastly, we cannot rule out the possibility that the moderation analysis was underpowered. The category consisting of persons unable to walk 2 km was significantly smaller than the other walking ability categories, and the resilience scores clustered toward the lower end of the scale in this group. Hence, testing the moderation effect at relatively high values of resilience may have been difficult.

### Limitations and strengths

This study has its limitations. Notable is that the data collection methods were different at baseline (computer-assisted personal interviewing conducted at participants’ homes) and follow-up (postal questionnaire), which may cause some bias regarding longitudinal analyses. Some downsides relate particularly to the postal questionnaires used amid the COVID-19 pandemic, as it is possible that the participants have misunderstood the questions. In addition, we do not know if it was the intended participant or some proxy, for example a spouse, who filled in the questionnaire. However, it is plausible that the effects of these limitations on the results are minor. It is also possible that selection bias has influenced the results to some extent. Although the sampling method was probability based, the participants of this longitudinal study were rather healthy and high-functioning whereas participants who dropped-out between baseline and follow-up were a little older and in poorer health. Hence, the findings of this study may present an underestimation of the actual influence of walking difficulty on active aging during this pandemic. Finally, it must be noted that although the study design was longitudinal, we cannot draw conclusions on causal relations and rule out the possibility that it was not COVID-19 or related measures that explain the associations found between walking difficulty, resilience, and active aging.

The strengths of the study are that, to the authors’ best knowledge, this was the first study to report on the predictors of decline in leading an active life during the COVID-19 pandemic compared to a situation 2 years before—a research need that has been called for (Rantanen et al. 2020). Other strengths of this study include comprehensive baseline data, a high response rate, and only little missing data. Furthermore, the follow-up data were collected during the early phases of the COVID-19 pandemic when there were rather few cases in the study area (102 confirmed infections in the study area, i.e., in the Central Finland Central Hospital district, population 253,000 inhabitants, 21 municipalities). Hence, the negative associations observed here were likely due to activity restrictions and social distancing, not due to the disease itself.

### Conclusions

In conclusion, the findings of this study show that mobility limitations exposed to greater declines in leading an active life during the early-phase COVID-19 pandemic, and that higher self-reliance on own abilities to overcome challenges alleviated this decline among persons with early-phase walking difficulties but not among those with more severe mobility limitations. Compared to previous suggestions on the contribution to resilience, i.e., the ability to positively adapt to adversity, on active aging among persons with early-phase mobility decline, the present study extends to the context of social distancing and reports on the benefits of resilience in the face of a unique, tangible adversity that is similar and present for all respondents. Overall, these findings highlight the importance of promoting utilization of coping strategies that help support resilience, e.g., compensatory actions or social support (Rutter 2006), especially among older people with declining mobility. In the future, it would be interesting to investigate whether persons with higher resilience, regardless of walking difficulties, return to their initial level of active aging quicker than persons with lower resilience, or whether they resume the initial levels of activity in the first place when the pandemic is over.

## Data Availability

Pseudonymized data are available to external collaborators upon agreement on the terms of data use and publication of results. To request the data, please contact Professor Taina Rantanen (taina.rantanen@jyu.fi).
